# The Dawn of Precision Medicine in Pediatric Nephrology: Lumasiran and the Era of siRNA Therapies for Primary Hyperoxaluria Type 1

**DOI:** 10.3390/jpm16010015

**Published:** 2026-01-02

**Authors:** John Dotis, Maria Fourikou

**Affiliations:** Third Department of Pediatrics, Aristotle University of Thessaloniki, Hippokration Hospital, 54642 Thessaloniki, Greece; mfour@auth.gr

**Keywords:** primary hyperoxaluria type 1, Lumasiran, siRNA therapeutics, pediatric nephrology, precision medicine

## Abstract

Primary hyperoxaluria type 1 (PH1) is a rare autosomal recessive disorder that causes progressive renal failure, nephrolithiasis, and nephrocalcinosis in children. It is characterized by hepatic overproduction of oxalate. Conventional management, which involves combined liver–kidney transplantation, vitamin B6 supplementation, and intense hydration, does not address the underlying metabolic defect for most patients and it generally provides only supportive care. The first approved disease-modifying treatment for pediatric PH1 is Lumasiran, a small interfering RNA (siRNA) therapeutic. By specifically inhibiting the hepatic glycolate oxidase mRNA, Lumasiran lowers the production of oxalate at its origin. Along with fewer kidney stone events and stabilization of nephrocalcinosis, clinical trials (ILLUMINATE-A/B/C) showed significant decreases in urinary oxalate excretion. The most frequently reported adverse event is mild injection-site reactions, which are generally well tolerated. The molecular mechanism, pharmacokinetics, and clinical effectiveness of Lumasiran in children with PH1 are compiled in this review. We go over possible long-term safety concerns, the impact of early intervention on renal outcomes, and the function of siRNA therapies in pediatric precision medicine. Furthermore, we highlight Lumasiran’s importance as a model for targeted treatment in uncommon pediatric kidney diseases by considering it in the larger context of RNAi-based therapies. A paradigm shift in pediatric nephrology is signaled by Lumasiran, which changes the therapeutic approach from supportive care to precision, targeted medicine. Further research and empirical data will clarify its long-term advantages, the best ways to treat it, and the possible use of siRNA technologies for other genetic renal disorders.

## 1. Introduction

Precision medicine is reshaping modern clinical practice, shifting the therapeutic focus from generalized approaches to interventions tailored to a patient’s unique genetic, environmental, and biological profile [1,2]. This shift holds promise for pediatric nephrology, where many chronic kidney diseases stem from single-gene defects and have historically lacked treatments that address the underlying cause [3,4]. For decades, management in these conditions was largely supportive, aiming to slow progression rather than offer meaningful disease modification [5,6].

RNA-based therapeutics, including antisense oligonucleotides (ASOs) and small interfering RNAs (siRNAs), have emerged to bridge this gap. These molecules act as highly precise molecular “switches”, capable of silencing or modulating genes that drive disease processes previously considered untreatable. Advances such as N-acetylgalactosamine (GalNAc) conjugation for targeted hepatic delivery have transformed RNA therapies from theoretical constructs into clinically applicable tools, with several approved agents and many more in development across nephrology [7,8,9].

Primary hyperoxaluria type 1 (PH1) exemplifies the urgent need for such targeted therapies. This rare disorder results from hepatic absence of alanine–glyoxylate aminotransferase (AGXT) gene, leading to unchecked oxalate overproduction and rapid progression toward nephrocalcinosis, recurrent stone formation, and ultimately end-stage kidney disease. Historically, many patients required combined liver–kidney transplantation, a high-risk and resource-intensive intervention [10,11].

A fundamentally different therapeutic strategy is introduced by siRNA technology, acting early in the metabolic pathway before the consequences of AGXT deficiency manifest. Lumasiran, the first approved siRNA for PH1, targets the hydroxyacid oxidase 1 transcript to reduce glyoxylate availability and thereby limit oxalate formation. Clinical trials have consistently shown large reductions in urinary and plasma oxalate, fewer stone events, and stabilization of kidney function across diverse age groups. Its targeted delivery and favorable safety profile have set a new benchmark for RNA therapeutics in pediatrics [12,13,14]. The introduction of Lumasiran into clinical practice confirms the potential of siRNA technology to serve as a platform for individualized therapy, realizing the goal of precision medicine for the treatment of genetic diseases.

The rapid evolution of this field is now influencing the broader landscape of nephrology. A growing number of RNA-based approaches are being explored for metabolic, immunologic, and genetic kidney disorders, reflecting a shift toward therapies that directly engage with the molecular basis of disease. For children, this approach is particularly compelling, as many monogenic kidney disorders are present early in life and may respond optimally to interventions delivered before irreversible injury occurs. However, application in the pediatric population requires careful consideration of developmental pharmacokinetics, immune responses, and long-term safety monitoring [15,16,17].

Lumasiran demonstrates how precision molecular therapeutics can be successfully translated into clinical practice and offers a roadmap for future RNA-driven programs. Continued progress will depend on integrating advanced RNA design with routine genetic diagnostics and learning health systems that support equitable, personalized care for all children with kidney disease [18,19,20].

This review fills a clear gap by providing a pediatric-focused synthesis of Lumasiran and its clinical development. We integrate child-specific challenges, including developmental pharmacokinetics, immune maturation, and the implications of lifelong therapy, with a comprehensive overview of the full ILLUMINATE program and its long-term extensions. We also incorporate emerging real-world experience and place Lumasiran within the evolving landscape of precision medicine and RNA-based therapeutics, while considering the future role of AI-supported drug development. Together, this review offers a coherent and up-to-date guide tailored to the needs of pediatric nephrology.

## 2. Pathophysiology of Primary Hyperoxaluria Type 1

PH1 is an autosomal recessive metabolic disorder caused by pathogenic variants in the AGXT gene, which encodes the hepatic enzyme alanine glyoxylate aminotransferase (AGT). Proper targeting of AGT to the peroxisome is essential for the conversion of glyoxylate to glycine. Many pathogenic variants, such as Gly170Arg or the splice-site change c.9431GT, impair this process by reducing catalytic activity, destabilizing the protein, or redirecting it to the mitochondria. As a result, little or no functional AGT remains available in the hepatocyte, and glyoxylate is allowed to accumulate [5,6,21].

Excess glyoxylate within the hepatocyte is rapidly metabolized by lactate dehydrogenase into oxalate. Under physiological conditions, this pathway contributes only a small proportion of oxalate production. In PH1 it becomes the dominant metabolic route. The shift in the glyoxylate pool toward oxalate formation creates a continuous and unregulated source of this toxic product. Because oxalate cannot be further degraded in humans, complete reliance on renal excretion follows. Gradual supersaturation of the tubular fluid promotes the intrarenal deposition of calcium oxalate, initiating tubular injury and interstitial inflammation [9,22].

A broad clinical spectrum arises from this metabolic disturbance. In some children, recurrent episodes of nephrolithiasis dominate the presentation and may be accompanied by flank pain or hematuria. However, in infancy, diffuse nephrocalcinosis often develops rapidly, leading to an early decline in renal function. Once glomerular filtration begins to fall, oxalate escapes into the systemic circulation. Soft tissues, including the myocardium, bones, retina, skin, and vascular walls, gradually accumulate oxalate crystals. This systemic deposition process, termed oxalosis, can involve multiple organs and present with cardiomyopathy, conduction abnormalities, bone pain, pathological fractures, anemia, and peripheral neuropathy. Transition to systemic oxalosis signifies an advanced stage of disease with a markedly poor prognosis [20,21,23].

Conventional therapy focuses on limiting intratubular oxalate concentration. High fluid intake throughout the day serves to maintain high urine flow rates, while citrate therapy helps to inhibit crystal aggregation. Pyridoxine can improve metabolic control in a minority of patients, particularly in those with specific AGXT variants that retain some responsiveness to cofactor supplementation. Unfortunately, in most individuals, pyridoxine has minimal influence on endogenous oxalate production. When renal function deteriorates, dialysis cannot match the overwhelming hepatic generation of oxalate. Even daily or intensified regimens fall short of achieving a negative oxalate balance [6,19].

For decades, combined liver and kidney transplantation represented the only way to correct both the metabolic defect and the consequences of renal failure. Replacement of the liver restores AGT activity, whereas the kidney transplant provides functional excretory capacity. Significant perioperative risks, the lifelong burden of immunosuppression, limited organ availability, combined with the poor feasibility of transplantation in infants continue to pose substantial barriers. Kidney transplantation alone, with the metabolic defect left uncorrected, inevitably leads to rapid graft loss due to recurrent oxalate deposition [6,19,24].

The pathophysiology of PH1 highlights a central principle. At its core lies a discrete hepatic enzymatic deficiency, while all renal and systemic manifestations arise secondarily from excessive oxalate production. Recognition of this mechanism paved the way for modern RNA-based therapeutics that interfere earlier in the metabolic pathway by reducing the formation of glyoxylate itself. By targeting upstream enzymatic steps, contemporary agents provide the first opportunity to lower oxalate at its molecular origin and to prevent progressive renal damage, an approach that closely aligns with the principles of precision medicine by directly addressing the underlying molecular defect [9,22,25].

## 3. Mechanism of RNA Interference and siRNA Therapeutics

Given that PH1 manifests early in life and progresses rapidly, a mechanistic understanding of siRNA function is directly relevant to pediatric therapeutic decision-making. RNA interference represents a highly conserved biological process through which cells regulate gene expression at the post-transcriptional level. Central to this mechanism is the selective degradation of messenger RNA molecules, allowing the cell to silence specific genes with remarkable precision. At the heart of this pathway lies a coordinated sequence of events involving the Dicer and Argonaute enzymatic proteins, together with additional protein partners that assemble into the RNA-induced silencing complex. Through this machinery, short double-stranded RNA molecules are converted into active regulatory elements capable of suppressing target transcripts [26,27].

**Figure 1:**
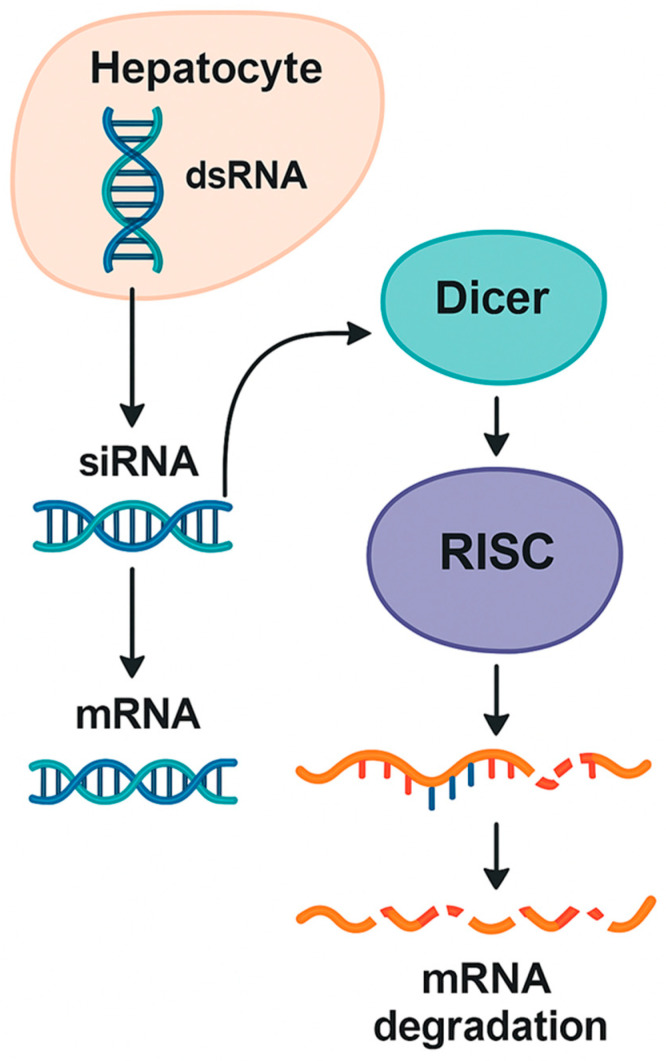
Overview of the RNA interference mechanism in hepatocytes.

Within the cytoplasm, exogenous or endogenously produced short RNA duplexes, typically 21 to 23 nucleotides in length, are recognized by the enzyme Dicer. Acting as a molecular sculptor, Dicer cleaves longer precursor RNAs into uniform fragments that are then passed on to Argonaute proteins. The resulting duplex is loaded into the RNA-induced silencing complex. During this process, one strand, known as the passenger strand, is removed and degraded. The other, called the guide strand, remains bound to Argonaute and dictates target recognition. By pairing with complementary sequences on specific messenger RNA molecules, the guide strand steers the ribonuclease activity of Argonaute toward the correct transcript (Figure 1) [28,29].

A schematic depiction of how double-stranded RNA precursors are processed by Dicer into short RNA duplexes that are loaded onto the RNA-induced silencing complex. After guide strand selection, the complex binds complementary messenger RNA targets, leading to their sequence-specific degradation or translational repression, thereby enabling targeted gene silencing within hepatocytes [26,27,28].

Once the guide strand engages its target, the RNA-induced silencing complex (RISC) induces messenger RNA degradation or translational repression. Complete complementarity between the guide strand and its target typically results in direct cleavage of the messenger RNA. Partial complementarity, in contrast, may lead to inhibition of translation without immediate degradation. Through these carefully orchestrated interactions, the cell can fine-tune gene expression with considerable specificity [25,30]. This inherent flexibility allows RNA interference to fine-tune gene expression and contributes to the predictable pharmacodynamic behavior seen with therapeutic siRNAs.

Therapeutic applications of siRNA take advantage of this natural process. By delivering synthetic RNA duplexes that mimic the products of Dicer, researchers can direct RISC to silence virtually any gene of interest. This approach offers several advantages, combining high specificity, because the sequence of the guide strand determines the target, with substantial programmability that allows the rapid design of molecules directed against new genes. In addition, it acts catalytically, since a single guide strand can recruit the RISC to multiple messenger RNA molecules. These properties form the basis for using siRNAs as mechanism-based, precision tools in metabolic and genetic diseases [4,9].

Lumasiran provides a representative example of this therapeutic strategy in clinical practice. Instead of targeting AGXT directly, the drug binds to messenger RNA derived from the hydroxyacid oxidase 1 gene, which encodes glycolate oxidase. Inhibition of this enzyme reduces the formation of glyoxylate, thereby decreasing the metabolic flux toward oxalate. By acting upstream in the biochemical pathway, Lumasiran prevents the buildup of toxic intermediates, thereby protecting the kidney and other organs from long-term injury. Importantly, hepatocyte-specific delivery is achieved through conjugation of the siRNA molecule with triantennary N-acetyl-galactosamine, a ligand that binds the asialoglycoprotein receptor on hepatocytes and thereby ensures selective uptake while minimizing systemic exposure. The success of Lumasiran in clinical trials underscores the remarkable potential of siRNA therapeutics for treating metabolic disorders. This targeted design illustrates how molecular engineering can be directly translated into clinically meaningful metabolic correction in children with PH1 [10,12,31].

Comparison with other RNA-based technologies highlights the unique advantages of siRNA. Antisense oligonucleotides bind to complementary messenger RNA sequences through single-stranded interactions and frequently rely on the recruitment of endogenous ribonucleases to degrade the target transcript. Their mechanism can be highly effective but often depends on chemical modifications to improve stability and tissue uptake. MicroRNAs represent endogenous regulators that modulate gene networks through partial complementarity. Although they can influence multiple targets simultaneously, this broad activity may limit their therapeutic specificity. In contrast, siRNA molecules operate through a highly specific duplex-mediated mechanism, achieve precise targeting, and rely on a powerful and catalytic endogenous pathway [4,7,8].

Taken together, RNA interference provides a biologically elegant and clinically versatile platform for gene silencing. The success of siRNA therapeutics in PH1 demonstrates how manipulation of this pathway can address the fundamental cause of a previously untreatable disease. As these technologies evolve, their mechanistic precision aligns naturally with the goals of pediatric precision medicine, particularly for monogenic kidney disorders [9,29].

A concise overview of RNA interference is included here because it provides the mechanistic foundation necessary to understand how Lumasiran was engineered and to clarify how these molecular principles translate into therapeutic decision-making in children with PH1.

## 4. Clinical Development of Lumasiran: Evidence from the ILLUMINATE Trials

The clinical development of Lumasiran followed a structured program of phase I through phase three studies collectively known as the ILLUMINATE trials (Table 1).

These investigations evaluated the safety, pharmacodynamics, and clinical efficacy of the drug in infants, children, adolescents, and adults with PH1. Through gradual expansion from tightly controlled pivotal cohorts to broad real-world populations, this program provided a comprehensive view of how hepatic inhibition of glycolate oxidase translates into meaningful biochemical and clinical benefits. Across all trials, the reduction in urinary and plasma oxalate concentrations remained the central therapeutic endpoint, reflecting the core metabolic defect in this disease [10,13,22].

### 4.1. ILLUMINATE a Trial

The pivotal phase three study, ILLUMINATE A, was a randomized, placebo-controlled trial that enrolled patients aged 6 years and older with preserved renal function. Participants received Lumasiran or placebo for 6 months, followed by an open-label extension. The primary endpoint was the percent change in 24 h urinary oxalate excretion from baseline to month 6. Secondary endpoints included the proportion of patients who achieved normal or near normal urinary oxalate, changes in urine oxalate to creatinine ratio, and assessment of safety outcomes [10,32].

A rapid and clinically meaningful response emerged, with mean urinary oxalate excretion declining by approximately 65–70% compared with placebo. Most patients reached near normal levels during the first few months of treatment, while biochemical control persisted throughout the extension period. Oxalate concentrations in plasma also decreased, providing additional evidence that upstream metabolic inhibition effectively reduces systemic oxalate burden. Clinical observations complemented the biochemical findings, revealing that fewer stone events were recorded, nephrocalcinosis scores stabilized, and quality-of-life measures improved in many participants [10,22].

Safety assessments in ILLUMINATE-A revealed a consistent and favorable profile, with injection-site reactions occurring most frequently, although the majority were mild and transient. No treatment-related serious adverse events were observed, and no patient discontinued therapy for safety reasons, underscoring the overall tolerability and favorable safety profile of the regimen. The combined efficacy and safety signal from this study established Lumasiran as an effective disease-modifying treatment for patients with adequate renal function [10,15].

### 4.2. ILLUMINATE B Trial

A separate phase III study, ILLUMINATE B, evaluated Lumasiran in children younger than 6 years of age, including infants as young as 6 months. These patients represented a crucial target group because early childhood is often the most aggressive phase of PH1. In this open-label study, the urinary oxalate to creatinine ratio served as the primary endpoint because 24 h urine collection is difficult in very young children. Treatment was administered monthly for the initial loading phase and then every 3 months thereafter [12,31].

The magnitude of biochemical improvement in ILLUMINATE-B surpassed that seen in older children and adults; notably, the mean urinary oxalate-to-creatinine ratio fell by 89% from baseline. The reduction occurred quickly, typically within the first 8–12 weeks, and remained stable over the full 12-month evaluation period. Oxalate concentrations in plasma also declined substantially. Additionally, imaging studies demonstrated stabilization or improvement of nephrocalcinosis in many infants, an important achievement given the high risk of irreversible renal damage during early childhood [12,17].

The safety profile in this younger cohort was consistent with that observed in ILLUMINATE-A, with injection-site reactions constituting the most common and generally mild adverse events, while no treatment-related discontinuations or systemic toxicities were reported. These findings demonstrate substantial biochemical improvement in early childhood; however, any potential effect on long-term renal preservation remains theoretical. The ILLUMINATE-B study was not designed to assess renal survival, and definitive evidence regarding nephron preservation is still lacking [31,32].

### 4.3. ILLUMINATE C Trial

The ILLUMINATE C study expanded the investigation to patients with advanced renal impairment, including those requiring dialysis. This open-label trial provided insight into the pharmacodynamics of Lumasiran in individuals with limited renal clearance of oxalate, a population that historically experienced the highest risk of systemic oxalosis. Patients received the same loading and maintenance dosing used in earlier studies [13,14].

Despite severe reductions in glomerular filtration rate, treatment produced meaningful reductions in plasma oxalate. The decline averaged around 30–40% in patients on dialysis and exceeded 50% in those with residual kidney function. These benefits were clinically relevant because lower systemic oxalate accumulation may reduce crystal deposition in soft tissues and improve the outlook for eventual kidney transplantation. Importantly, the drug was well tolerated even in this medically fragile population [13,20].

Emerging post-marketing pharmacovigilance data and recent real-world pediatric registries provide a clearer understanding of Lumasiran’s performance outside controlled trials [34,35]. These cohorts show that children do not respond uniformly to therapy, with many achieving substantial reductions in urinary oxalate, whereas others experience only partial or minimal improvement despite adequate glycolate oxidase inhibition. Τhis variability, more evident in routine practice than in trials, highlights the need for balanced interpretation and structured long-term monitoring. Real-world experience also indicates that factors such as adherence, comorbidities, and individual metabolic differences may influence outcomes, supporting more individualized follow-up. Overall, these observations underscore that real-world effectiveness may differ from trial-based expectations and reinforce the importance of expanding registries to better define long-term renal and systemic outcomes in pediatric PH1.

### 4.4. Long-Term Data

Long-term insights come from the extended follow-up of the ILLUMINATE studies, which now exceed 54 months of observation in many patients. The sustained reduction in urinary and plasma oxalate has remained striking. Most patients continued to exhibit reductions of 60–70% percent beyond 4 years of therapy. No evidence of tachyphylaxis or loss of efficacy has been observed, indicating durable metabolic control [15,33].

Safety findings during extended follow-up remained consistent with earlier phases of development. There has been no emergence of new safety signals, no drug-related hepatic toxicity, and no concerns regarding immunogenicity. Injection site reactions gradually decreased over time as patients and caregivers gained experience with administration. These long-term results reinforce the reliability of siRNA-mediated metabolic inhibition as chronic therapy [18,33]. Nevertheless, important safety uncertainties remain, particularly for children who may require lifelong treatment. Off-target gene silencing, although not detected in current trials, represents a theoretical risk inherent to all siRNA platforms, together with the cumulative impact of repeated dosing over several decades is unknown. No data beyond approximately four to five years of exposure are available and the absence of long-term developmental, hepatic, or immune surveillance highlights the need for ongoing pharmacovigilance and structured pediatric registries.

It should also be noted that available studies provide limited data on hard renal outcomes such as progression to end-stage kidney disease, timing of transplantation, or long-term quality-of-life measures. The current evidence base is dominated by biochemical endpoints, and definitive renal survival data remains an important unmet need for future research.

### 4.5. Renal Function Preservation

Perhaps the most clinically meaningful observation across the ILLUMINATE program is the stabilization of renal function. While the studies were not primarily powered to evaluate hard renal endpoints, trends toward slower decline in estimated glomerular filtration rate have appeared repeatedly. The prevention of ongoing oxalate overproduction reduces tubular injury and mitigates inflammatory and fibrotic processes within the kidney. In infants treated early, stabilization of nephrocalcinosis strongly suggests that early intervention may preserve nephron mass and protect long-term renal outcomes [12,22].

Taken together, the ILLUMINATE trials demonstrate that Lumasiran offers rapid, robust, and sustained metabolic control across all ages and disease stages. The accumulated evidence supports a paradigm in which early and continuous reduction in hepatic oxalate production can preserve kidney health, limit the need for intensive dialysis, and improve the feasibility of successful transplantation when required [10,12].

## 5. Beyond Lumasiran: The Expanding siRNA Landscape

**Figure 2:**
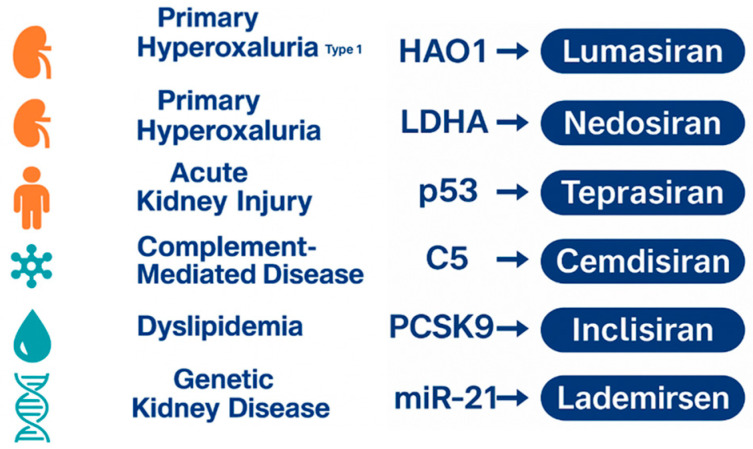
Overview of the emerging siRNA therapeutic pipeline across major disease areas.

The clinical success of Lumasiran has accelerated the development of other siRNA therapeutics across nephrology and related fields (Figure 2).

A visual summary of representative disease categories currently targeted by experimental siRNA therapies. The figure illustrates key molecular targets under investigation, reflecting the expanding application of RNA interference beyond primary hyperoxaluria into metabolic, inflammatory, cardiovascular, and genetic kidney disorders [25,30,36].

In this section, agents with direct relevance to pediatric nephrology or metabolic pathways related to PH1 are presented in greater detail, whereas others are discussed more briefly to situate Lumasiran within the broader therapeutic landscape.

Although PH1 offered a clear metabolic target, many additional pathways in kidney disease can be modulated through selective post-transcriptional gene silencing. The expanding siRNA pipeline reflects growing confidence in hepatocyte targeting, improved chemical stability, and a more mature understanding of GalNAc-mediated delivery [4,9].

Among the most advanced candidates is Νedosiran, a siRNA that targets lactate dehydrogenase A. By inhibiting this terminal enzyme in the oxalate-producing pathway, nedosiran aims to treat not only PH1 but also hyperoxaluria type 2 (PH2) and type 3 (PH3). Instead of correcting a single enzymatic defect, it acts at a metabolic convergence point shared across all forms of the disease. Early clinical studies demonstrated marked reductions in urinary oxalate and favorable tolerability. Data from the PHYOX clinical program further supported its potential, with long-term extension studies showing sustained reductions in urinary oxalate and an acceptable safety profile, while population pharmacokinetic modeling provided dosing guidance for pediatric patients [37,38]. However, results from later-phase trials revealed variable responses in patients with PH3, reflecting the more complex nature of glyoxylate handling in this subtype. Even so, Nedosiran remains a promising agent for a broader spectrum of hyperoxaluric disorders, particularly for those who lack pyridoxine-responsive genotypes [37,38].

The therapeutic reach of siRNA technology extends well beyond metabolic stone disease. Teprasiran represents a distinct application, as it downregulates the expression of p53 to prevent acute kidney injury in high-risk surgical patients. The rationale stems from the central role of p53 in apoptosis triggered by ischemic or toxic stress. By temporarily reducing p53 activation during periods of profound ischemia and reperfusion, Teprasiran aims to protect tubular epithelial cells and reduce the incidence of postoperative acute kidney injury. Phase II data show encouraging trends toward lower rates of early kidney injury after cardiothoracic procedures, although larger trials are needed to confirm clinical benefit [36,39].

Other siRNA therapies explore immunologic and systemic cardiovascular targets with clear implications for nephrology. Cemdisiran, which silences complement component C5, has undergone investigation in IgA nephropathy and other complement-mediated diseases. Complement overactivation can accelerate glomerular injury in a range of immune glomerulopathies, and the ability to suppress hepatic C5 synthesis offers a sustained alternative to monoclonal antibody therapy. Early findings suggest significant reductions in serum C5 levels with a tolerable safety profile [40,41].

Another agent, Inclisiran, has been approved for the treatment of dyslipidemia in adults through its inhibition of PCSK9, a regulatory molecule that governs hepatic LDL receptor turnover. Although primarily a cardiovascular drug, its long dosing interval and consistent LDL reduction raise interest in adults and adolescents with genetic dyslipidemias that contribute to chronic kidney disease progression [42,43].

The exploration of siRNA technology also reaches into the field of genetic kidney disease. Lademirsen, designed to inhibit microRNA 21, has been investigated in Alport syndrome. MicroRNA 21 is implicated in fibrotic signaling pathways that drive progressive glomerulosclerosis. By reducing microRNA 21 activity, Lademirsen aims to modulate the downstream pathways that lead to interstitial fibrosis. Animal models showed notable improvement, but early clinical translation provided mixed results, underscoring the complexity of fibrotic pathways and the potential need for combination approaches rather than single-target modulation [9,44].

It is important to note that most emerging siRNA therapies have been developed and tested predominantly in adult populations, with agents such as Teprasiran, Cemdisiran, Inclisiran, and Lademirsen supported almost entirely by adult-focused trials. As a result, their relevance for children and adolescents with kidney disease remains uncertain. In contrast, Lumasiran and Nedosiran are the only siRNA-based therapies that have undergone dedicated pediatric evaluation, and both now hold regulatory approval for use in children, representing the first successful translation of RNA interference technology into routine pediatric nephrology. As the field evolves, ongoing safety surveillance and long-term registries will be essential to guide their use across the pediatric age spectrum [16,18].

Although current siRNA therapeutics rely primarily on hepatocyte-directed GalNAc delivery, several early-phase strategies aim to enable kidney-specific targeting. Experimental approaches include peptide-based ligands designed to bind megalin or cubilin in the proximal tubule, nanoparticle formulations engineered to avoid rapid filtration, and chemically modified RNA conjugates capable of enhancing tubular uptake. These renal-targeted platforms remain in preclinical development, but they highlight potential pathways for overcoming the major anatomical and physiological barriers to siRNA delivery within the kidney [3,7,9].

The rapid expansion of the siRNA landscape inevitably raises questions regarding pediatric application. Children present unique considerations in pharmacokinetics, tissue maturation, immune tolerance, and long-term safety. Lifelong hereditary diseases require decades of exposure, mandating careful evaluation of off-target effects and immune reactions to repeated dosing. Nevertheless, several features of siRNA therapy favor pediatric use. Their liver-specific uptake limits systemic exposure, dosing intervals are long, and their mechanism is reversible rather than permanent. Inherited metabolic diseases, many of which are present in infancy or early childhood, are particularly suited to early intervention strategies that prevent irreversible organ injury [34,45].

Despite these promising features, the broader clinical and economic value of siRNA therapies has not yet been fully defined. Formal cost-effectiveness analyses and long-term comparative outcome studies remain limited, making it difficult to determine how these agents will be incorporated into routine pediatric care on a population level [9,22,24].

This harmonized structure ensures consistency across the section while maintaining emphasis on siRNA therapies with the most direct clinical implications for pediatric kidney disease.

## 6. Challenges and Future Perspectives

Despite the clinical success of Lumasiran and the rapid expansion of RNA-based therapies in nephrology, several challenges continue to shape the development and future implementation of siRNA therapeutics. Many of these challenges relate to delivery, safety, accessibility, and the ability to translate molecular precision into broad clinical benefit, particularly for pediatric patients [9,30].

One of the principal scientific hurdles concerns the delivery of siRNA molecules to the correct tissue. Hepatocyte targeting has become highly efficient through GalNAc-based delivery platforms, which exploit receptor-mediated uptake unique to the liver. This strategy works well for diseases in which the primary metabolic defect resides in the liver, such as the hyperoxalurias. However, most genetic kidney diseases arise in renal tubular or glomerular cells, which do not possess comparably efficient or specific uptake mechanisms for siRNA delivery. Nanoparticle-based carriers, lipid vesicles, and engineered peptides offer potential solutions. Even so, many barriers remain, including rapid degradation in circulation, filtration through the glomerulus, and inefficient entry into highly specialized renal epithelial compartments. Future progress will depend on the development of vectors that can bypass these structural barriers and deliver siRNA precisely to renal targets without provoking toxicity [26,36].

Another major challenge relates to cost and accessibility. These therapeutics are sophisticated nucleic acid–based biologics that require highly specialized processes for synthesis, purification, and formulation, all of which contribute substantially to production expense. As a result, manufacturing costs remain high, and affordability is limited for many health systems. Global access becomes even more challenging in rare diseases, where very small patient populations make it difficult for developers to recover high research and development costs. Strategies to lower production expenses include enabling biosimilar development once patents expire, while the establishment of international funding mechanisms will be crucial to ensure equitable access [21,29].

Long-term safety remains a central concern, particularly for pediatric populations who may require lifelong therapy. Although hepatocyte-targeted siRNA molecules have shown excellent tolerability, potential risks cannot be fully excluded. Immune activation through innate sensing pathways, unintended silencing of transcripts with partial sequence complementarity, and cumulative off-target effects represent key safety considerations. Continuous postmarketing surveillance, long-term registries, and mechanistic studies are essential to evaluate how decades of siRNA exposure influence organ maturation and immune development. The reversible nature of RNA interference offers inherent safety advantages compared with permanent genome editing, yet vigilance remains necessary [9,27].

Regulatory oversight into pediatric use presents an additional layer of complexity. Children differ substantially from adults in pharmacokinetics, organ maturation, and immune reactivity. Regulatory agencies increasingly require pediatric investigation plans that address dosing, long-term follow up and developmental toxicity. Designing randomized trials in rare pediatric disorders remains challenging because of small sample sizes and significant phenotypic heterogeneity. Adaptive designs, real-world evidence, and global collaborative networks may help overcome these constraints [2,6].

**Figure 3:**
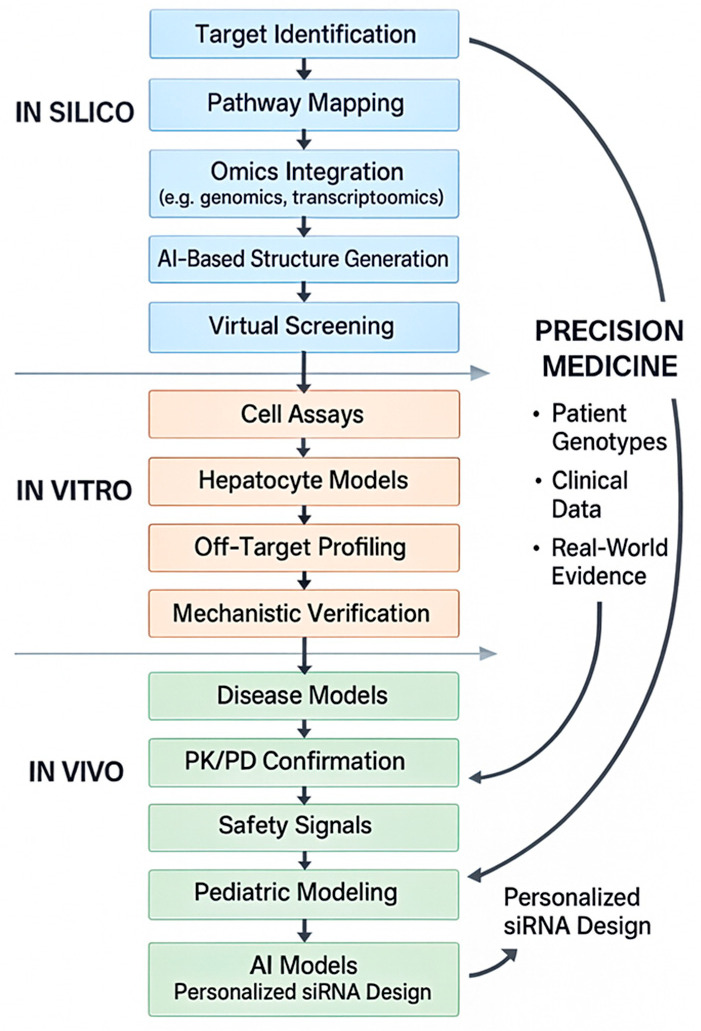
Integrated in silico, in vitro, and in vivo pipeline for AI-enabled design of precision RNA therapeutics.

Artificial intelligence (AI) is also emerging as a transformative force in the future of RNA-based therapeutics (Figure 3).

A full-text schematic illustrating how AI-driven in silico approaches integrate with in vitro validation, in vivo modeling, and patient-specific genomic data to support the development of precision RNA therapeutics in pediatric nephrology [46,47,48].

Recent advances show that AI systems can identify previously unrecognized molecular targets, design and optimize candidate molecules, and predict pharmacologic behavior with unprecedented speed. Instead of relying on traditional stepwise drug discovery pipelines, AI-driven platforms can screen millions of chemical structures, prioritize those with favorable biological properties, and simulate safety profiles before laboratory synthesis even begins. AI has also been used to discover new antimicrobial agents and to repurpose existing drugs for unexpected therapeutic indications, demonstrating the versatility of these computational tools. These developments illustrate how AI may accelerate RNA therapeutic discovery, reduce cost, and support individualized treatment strategies [46,47,48].

The future of siRNA therapy in nephrology is likely to extend well beyond metabolic disorders. Many monogenic nephropathies, including nephronophthisis, autosomal recessive polycystic kidney disease, and Alport syndrome, involve pathways that could potentially be modulated through RNA-based intervention. Improved delivery platforms and increasingly sophisticated computational discovery tools may eventually allow siRNA molecules to reach tubular, interstitial, and glomerular compartments with sufficient precision to achieve meaningful disease modification [9,44,45].

Taken together, the challenges facing siRNA therapeutics are substantial but not insurmountable. The foundation established by Lumasiran shows that precision molecular therapy can transform the natural history of pediatric kidney diseases with a defined metabolic origin. The convergence of RNA therapeutics and AI-driven drug discovery is expected to redefine future therapeutic development, bringing personalized treatments ever closer to routine pediatric nephrology practice [1,2]. While AI-driven design platforms have demonstrated measurable impact on siRNA optimization in oncology and metabolic drug development, there is currently no direct evidence that these tools have influenced siRNA therapeutics specifically within nephrology. Their anticipated role in pediatric kidney disease therefore remains prospective and forward-looking rather than evidence-based at this stage.

## 7. Conclusions

The emergence of RNA-based therapeutics marks the beginning of a new era in pediatric nephrology, representing the true integration of precision medicine into the management of hereditary kidney diseases. By targeting metabolic or genetic defects at their molecular origin, these therapies demonstrate a clear transition from symptomatic treatment toward interventions that are guided by specific pathogenic mechanisms. The ability to silence hepatic or renal pathways responsible for disease progression highlights the transformative potential of RNA interference as a long-term disease-modifying strategy [4,9].

This evolving therapeutic model illustrates how mechanism-based interventions can reshape clinical outcomes even in conditions historically managed only with supportive care or organ transplantation. As delivery technologies advance, RNA-based approaches are expected to expand into a broader spectrum of monogenic nephropathies, including disorders of tubular function, complement activation, fibrosis, and ciliopathies [1,45].

Despite these advances, important challenges remain. Genetic heterogeneity influences individual biochemical response and delayed diagnosis, which is still common in many regions, limiting the opportunity for timely intervention. Furthermore, access disparities and the high cost of RNA-based therapies may restrict availability in routine clinical practice. Recognizing these limitations is essential to situating Lumasiran and other siRNA agents within a realistic and equitable precision-medicine framework.

At the same time, the parallel rise of artificial intelligence offers complementary tools that accelerate target discovery, refine molecular design, and support the development of individualized treatment strategies. The convergence of RNA therapeutics, computational discovery platforms, and rapid genetic diagnostics positions pediatric nephrology to benefit substantially from these emerging technologies. Taken together, these developments suggest a future in which genetically informed and molecularly targeted therapies become an increasingly integral part of childhood kidney disease management.

## Figures and Tables

**Table 1:** Summary of the ILLUMINATE clinical trial program for Lumasiran.

Trial	Population	Design	Primary Endpoint	Key Efficacy Findings	Safety	Reference
ILLUMINATE-A	Patients ≥6 years with PH1 and preserved kidney function	Randomized, placebo-controlled (6 months) + extension	Change in 24 h urinary oxalate	65–70% oxalate reduction; many normalized levels; fewer stones	Mild injection-site reactions; no major safety issues	[10,32]
ILLUMINATE-B	Infants and children <6 years	Open-label multicenter trial	Urinary oxalate/creatinine ratio	89% reduction; stabilization/improvement of nephrocalcinosis	Mild local reactions; well tolerated	[12,17,31]
ILLUMINATE-C	Advanced CKD, including dialysis	Open-label	Plasma oxalate	30–50% reduction depending on residual function	Safe even in severe CKD; no dose-limiting toxicity	[13,14]
Long-term extension	Participants from all trials	Open-label long-term follow-up	Durability of response	Sustained 60–70% oxalate reduction over >4 years	Stable long-term safety; no emerging adverse signals	[15,18,33]

PH1: Primary Hyperoxaluria Type 1; CKD: Chronic Kidney Disease.

## Data Availability

No new data were created or analyzed in this study. Data sharing is not applicable to this article.

## Abbreviations

The following abbreviations are used in this manuscript:

PH1	Primary Hyperoxaluria Type 1
AGXT	Alanine Glyoxylate Aminotransferase gene
AGT	Alanine Glyoxylate Aminotransferase
HAO1	Hydroxyacid Oxidase 1 (Glycolate Oxidase)
GO	Glycolate Oxidase
LDH	Lactate Dehydrogenase
ASO	Antisense Oligonucleotide
siRNA	Small Interfering RNA
RNAi	RNA Interference
GalNAc	N-Acetylgalactosamine
RISC	RNA-Induced Silencing Complex
GFR	Glomerular Filtration Rate
CKD	Chronic Kidney Disease
